# A space–time analysis of the WikiLeaks Afghan War Diary: a resource for analyzing the conflict-health nexus

**DOI:** 10.1186/s12942-015-0022-8

**Published:** 2015-10-16

**Authors:** Andrew Curtis, Xinyue Ye, Kevin Hachey, Margaret Bourdeaux, Alison Norris

**Affiliations:** GIS, Health and Hazards Lab, Department of Geography, Kent State University, Kent, OH 44242 USA; College of Medicine, Ohio State University, Meiling Hall, 370 W 9th Ave, Columbus, OH 43210 USA; Division of Global Health Equity, Brigham and Women’s Hospital, 75 Francis Street, Boston, MA USA; Epidemiology and Medicine, College of Public Health, Ohio State University, 326 Cunz Hall, 1841 Neil Ave, Columbus, OH 43210-1351 USA

**Keywords:** Afghanistan, GIS, Spatio-temporal analysis, WikiLeak, Polio, Conflict health

## Abstract

**Background:**

Although it is widely acknowledged that areas of conflict are associated with a high health burden, from a geospatial perspective it is difficult to establish these patterns at fine scales because of a lack of data. The release of the “WikiLeaks” Afghan War Diary (AWD) provides an interesting opportunity to advance analysis and theory into this interrelationship.

**Methods:**

This paper will apply two different space time analyses to identify patterns of improvised explosive devices (IED) detonations for the period of 2004 to 2009 in Afghanistan.

**Results:**

There is considerable spatial and temporal heterogeneity in IED explosions, with concentrations often following transportation links. The results are framed in terms of a resource for subsequent analyses to other existing health research in Afghanistan. To facilitate this, in our discussion we present a Google Earth file of overlapping rates that can be distributed to any researcher interested in combining his/her fine scale health data with a similarly granular layer of violence.

**Conclusion:**

The release of the AWD presents a previously unavailable opportunity to consider how spatially detailed data about violence can be incorporated into understanding, and predicting, health related spillover effects. The AWD can enrich previous research conducted on Afghanistan, and provide a justification for future “official” data sharing at appropriately fine scales.

## Background

There are many direct and indirect health impacts for those living in conflict zones [17]. Public health and epidemiological research has previously suggested that severe externalities (natural/human cause disasters as well as conflicts) cause or exacerbate negative health outcomes [11, 32, 38]. Yet this relationship is rarely spatially studied in non-Western environments especially at local levels, primarily due to a lack of available fine scale data. One opportunity to address this gap has arisen with the release of “WikiLeaks,” and especially the Afghan War Diary (AWD) which offers 6 years of granular data in the form of Improvised Explosive Device (IED) locations, the weapon of choice for Taliban aligned forces in Afghanistan. These data can help enrich the existing body of health related research focused on Afghanistan, which though often makes reference to armed conflict, rarely incorporates *actual violence data* into their epidemiological models [10]. The purpose of this paper is twofold: to consider the fine-scale space–time patterns of the AWD. Secondly, to suggest how these data can be used to conduct secondary epidemiological investigations of previously described studies. In so doing the following research question is posed to illustrate the need for granular data:

Is the pattern of violence in Afghanistan, as captured by IED detonations in the AWD, spatially and temporally homogenous at the sub-district scale?

There have been multiple health-related studies focused on Afghanistan [22, 23, 27, 33], many of which also refer to the co-occurrence of violence [4]. However there are relatively few analyses of the nexus and when done usually conducted for broader aggregations such as the district or province [24]. Although identifying patterns at broad geographic scales are still revealing, finer scale data that reflect day-to-day activity, individual settlements and especially transportation corridors are needed if we are to advance a spatial theory of how violence might disrupt local health systems, lead to increased disease, and consequently how and where to target intervention.

There have been several spatially detailed studies of violence in Afghanistan and Iraq^1^ [3, 6, 7, 20, 21, 25, 39], though of relevance to this paper, is how there has been a lack of a standard reference conflict data source. Lacroix et al. [20] use the Information Management System for Mine Action (IMSMA), while Benigni and Furrer analyzed IED locations from Iraq Significant Activities (SIGACTS) III [7]. Medina and colleagues drew from the US National Counterterrorism Center’s (NCTC) Worldwide Incidents Tracking System (WITS), which although being a rich dataset of all attacks requires the researcher to use a gazetteer to geo-locate events [25]. Barker’s work on IEDs in Southern Afghanistan and Western Pakistan between 2002 and 2009 use kernel density estimation (KDE) to provide a detailed breakdown of violence across districts, showing how detonations had increased in number and lethality [6]. For this he synthesized three data sources (Maryland’s Global Terrorism Database (GTD), the National Counterterrorism Center’s Worldwide Incidents Tracking System (WITS) and Hazard Management Solution’s (HMS) TRITON Database), all of which contain information extracted from open source media, but again require the use of a gazetteer [6].

The WikiLeaks AWD offers an interesting alternative to these disparate sources, especially from a spatial perspective [39]. The AWD contains approximately 77,000 events covering the period 2004–2009, including the precise spatial location of all Improvised Explosive Devices (IEDs), the weapon of choice for insurgents in the region. Although NATO and other military forces have declined to confirm or deny the accuracy of these data, the New York Times, The Guardian, and Der Spiegel have all concluded that these data appear to be genuine, a finding validated in academia by comparison with other databases [39]. Though these data offer interesting possibilities for the spatial analysis of violence in the region, they also present a previously unavailable opportunity for epidemiologists.

### An example of why the conflict-health nexus is important: Polio in Afghanistan

An example of a disease which has been linked to regional violence is Poliomyelitis [1, 19]. Poliomyelitis is a viral disease spread through the fecal contamination of food or water sources. In 1988 the World Health Organization (WHO) proposed a strategy to combat the 400,000 global cases per year and effectively eradicate paralytic polio by the year 2000. Regional initiatives were successfully implemented throughout the 1990s [26]. By 2006, endemic polio was only found in four countries: Nigeria, India, Pakistan, and Afghanistan [35]. Obstacles to vaccination coverage, such as geographic barriers and relative cultural isolation, were overcome in India and Nigeria [5]. Disease reduction strategies in Pakistan and Afghanistan have not been as successful with the number of paralytic poliomyelitis cases actually rising in Afghanistan from a low of 4 in 2004 to a high of 80 in 2011 [30]; this despite WHO-UNICEF reported increases in the national vaccination coverage rate from 35 % in 2001 to 66 % in 2011. A major reason for this lack of success is the ongoing violence in the region. By 2010, in the southern region of Afghanistan (Nimruz, Helmand, Kandahar, Zabul and Oruzgan provinces), which is frequently mentioned in news stories with regard its levels of violence; only 26 % of children were adequately vaccinated against polio.

Conflict hinders vaccine programs in numerous ways: by disrupting refrigerated transportation of vaccine stocks, increasing dangers associated with medical center locations, especially in more turbulent or isolated environments and due to the distrust, fear and uncertainty of being associated with any “official” program. For this region in particular, health workers have become targets themselves, subject to murder, threats and staff abductions [34]. We analyzed the polio-violence nexus in Afghanistan at a coarse geographic scale by regressing 2761 Coalition force deaths reported by iCasualties.org between 2001 and 2011 against 264 laboratory-confirmed cases of acute flaccid paralysis caused by wild-type poliovirus. These case data originated from the World Health Organization’s (WHO) surveillance system which consists of a nationwide network of over 10,000 volunteers collecting acute flaccid paralysis counts, where in 80 % of cases lab work is used to identify wild-type poliovirus, vaccine-derived poliovirus, and related non-polio enteroviruses [36].^2^ The regression results which incorporated various lag components found a statistically significant pattern in the upward trends of both polio cases and coalition casualties. More specifically, for every unit increase in coalition casualties, polio cases increased by 0.129 2 years later (R^2^ = 0.802, p = 0.001).^3^ Or, an increase of approximately eight coalition casualties was associated with one additional case of polio. This finding is in keeping with other research; in Somalia where outbreaks occurred in 2011 and 2013, and Syria in 2011 there had been an escalation in violence approximately 2 years prior [8, 15].

Unfortunately without access to these disease data (or vaccine data) at a fine scale, or maps of violence at an equal granularity, this is where previous polio analyses in the region have stopped. Now, with the release of the AWD, we can perform more detailed analyses of the violence, leading to discussions with regards the spatial interaction between attack points, and immediately proximate health outcomes.

## Methods

The AWD contains multiple attributes, including the date of each event, the type of event (here we concentrate on IEDs), and latitude and longitude to eight decimal places. Text accompanying each record describes the number and type of causalities associated with the IED (civilian, coalition, or insurgent force death or injury), the time of the explosion, and other key features about the device. Although the number of casualties is available, we treat each IED event as a single entity. For our perspective this is a better measure of violence than casualties because the number of people in close proximity to the explosion is not reported. Thus there is no denominator to standardize this variable.^4^

Decisions have to be made with regards how to partition data in space and time for an analysis. For example, Benigni and Furrer [7] constrained their space time analysis of IEDs in Iraq to road segments, and to include a travel time variable from base to explosion as a distance measure, their decision in turn being influenced by the work of Lacroix et al. [20] who found that 47 % of all ERWs were no further than 1 km from a road. The concept of time is also important when considering violent attacks [7], not only because these events diffuse in space and time, but because key dates, policy changes, and even outside influence (such as importing IED “expertise” from Iraq), can influence patterns [6]. When considering insurgent attacks in Iraq between 2004 and 2009, Medina et al. [25] noted the importance of specific dates, but also found temporal sequences or patterns within the larger time column, such as an increased likelihood of a second attack within 8 weeks of the first [9, 25, 29]. Given the likely importance of both space and time in the AWD, we chose two different methods to investigate the patterns of IEDs; KDE which is a frequently used analysis and SaTScan analysis.

### Kernel density estimation

Kernel density estimation (KDE) is a commonly applied method to create “heat maps” in crime, epidemiological research [14, 16, 37], and other studies of violence in the region [2, 21]. All IEDs falling inside a kernel (a circular area extending away from a single location) become part of a density calculation. A quadratic function is used to reduce the influence of each IED as distance from the center of the kernel increases. Kernel size can dramatically change the final output map as the number of included IEDs varies; a small radius results in a more heterogeneous surface (at the extreme, each kernel only includes one IED), with more homogeneity occurring across the map as the kernel extends outwards (at the other extreme all IEDs are included in a single kernel). Although there are guidelines to suggest kernel size (the bandwidth), it is widely accepted that this is still a relatively arbitrary choice. However, understanding how varying kernel sizes can influence results is imperative [12, 18]. For the purpose of our analysis, and in order to give confidence in the stability of findings for any one bandwidth, several distances are chosen, though only the 50 km radius is described here. This kernel size yields a reasonable compromise between fine and coarse scale smoothing; this distance also falls in the middle of a set of KDE examples chosen by Lacroix and authors in a previous study of Afghanistan [20]. The output of a KDE is a raster map with each cell containing an IED density value. These cells are contoured for visualization purposes.

The AWD can be sorted by year, season, month, day or even time of day.^5^ Although different temporal aggregations were explored, results presented here are for IEDs per year which is the easiest aggregation to match with other research. When comparing KDE heat maps across the years, each map was classified separately for the data distribution of that year which allows for the comparison of general spatial patterns rather than intensities, even in years with lower numbers of IED explosions; in other words, the annual relative risk. As others have noted, the general trend in Afghanistan was an increase in IEDs of at least 40 % per year from 2002 to 2008, except for 2007. The largest single rise occurred in 2006 [6]. Therefore, applying the same class breaks would be problematic for years earlier in the sequence. The final output maps are classified into ten categories (approximating 10 % per color band) for each year. These classification breaks were used to determine our contour granularity. For the purposes of this paper, only the top five classifications for each year (the top 50 %) are overlaid on a composite map of all IEDs. This approach to visualizing spatio-temporal data using a relatively simple analytical approach has previously been applied by the authors for space–time sequences in epidemic disease [14].^6^

### Space–time disaggregation and SaTScan

KDE is a useful and easily applied technique to compare different time periods, though it is less useful at identifying temporal trends within the data. Alternatively SaTScan (v9.1.1) can reveal space and time patterns in locations collected across multiple years [13, 14, 31] as it explicitly considers spatial and temporal data point interconnections using a retrospective space–time permutation model with testing for statistical significance by a Monte Carlo simulation (with 999 simulation runs). Conceptually, a tube with a set kernel moves across the study space, with the body of the tube shrinking or expanding at every location as it identifies clusters of events (in this case IEDs). Although different kernel sizes were chosen, results reported here are for a maximum of 50 % of the population at risk being included.^7^ Just as with KDE, a decision has to be made as to the granularity of the time column. We chose an analytical unit of a day in order to reveal patterns potentially missed by month or 6 month aggregations.^8^^,^^9^

## Results

**Fig. 1:**
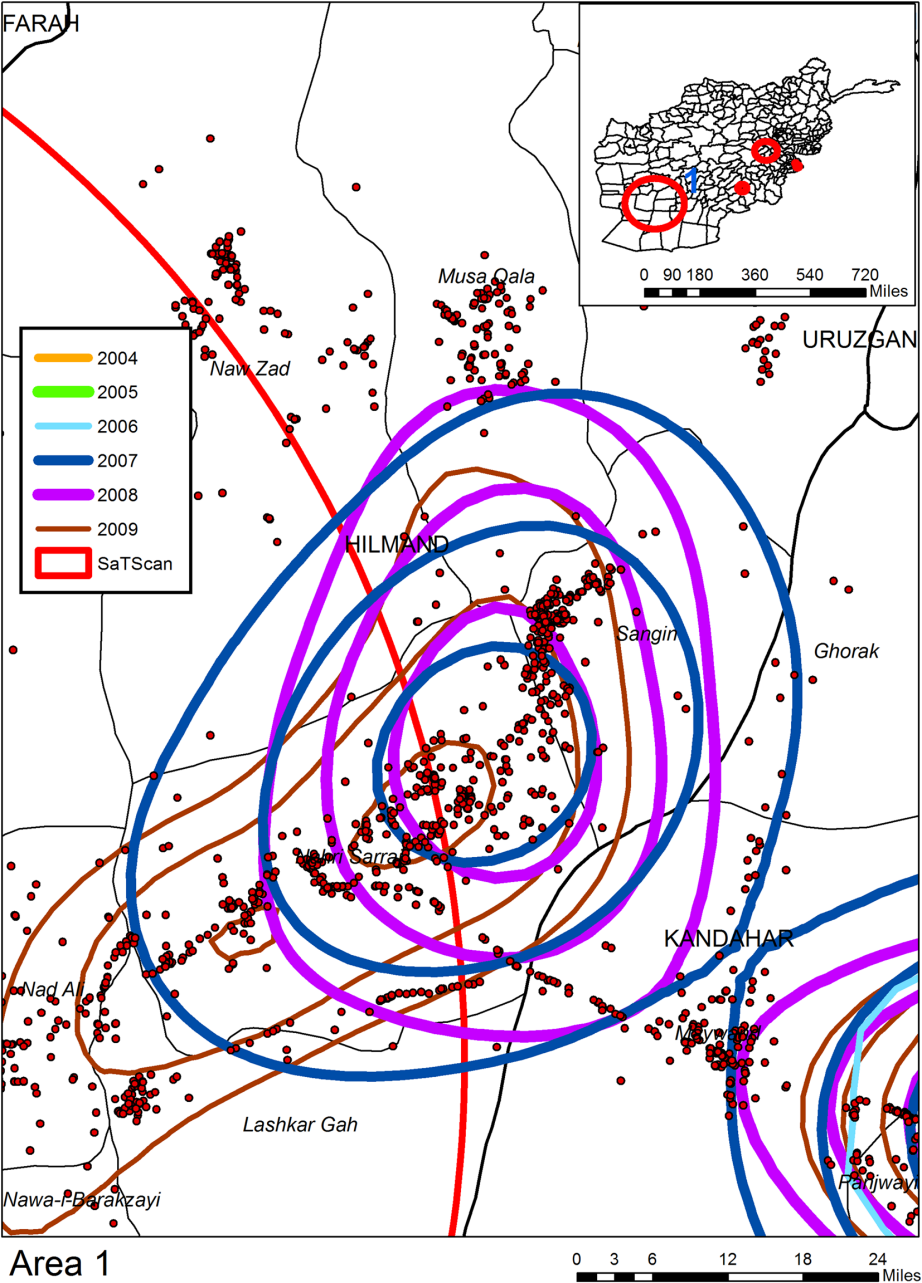
KDE and SaTScan analyses of IEDs for Area 1 (Helmand Province)

**Fig. 2:**
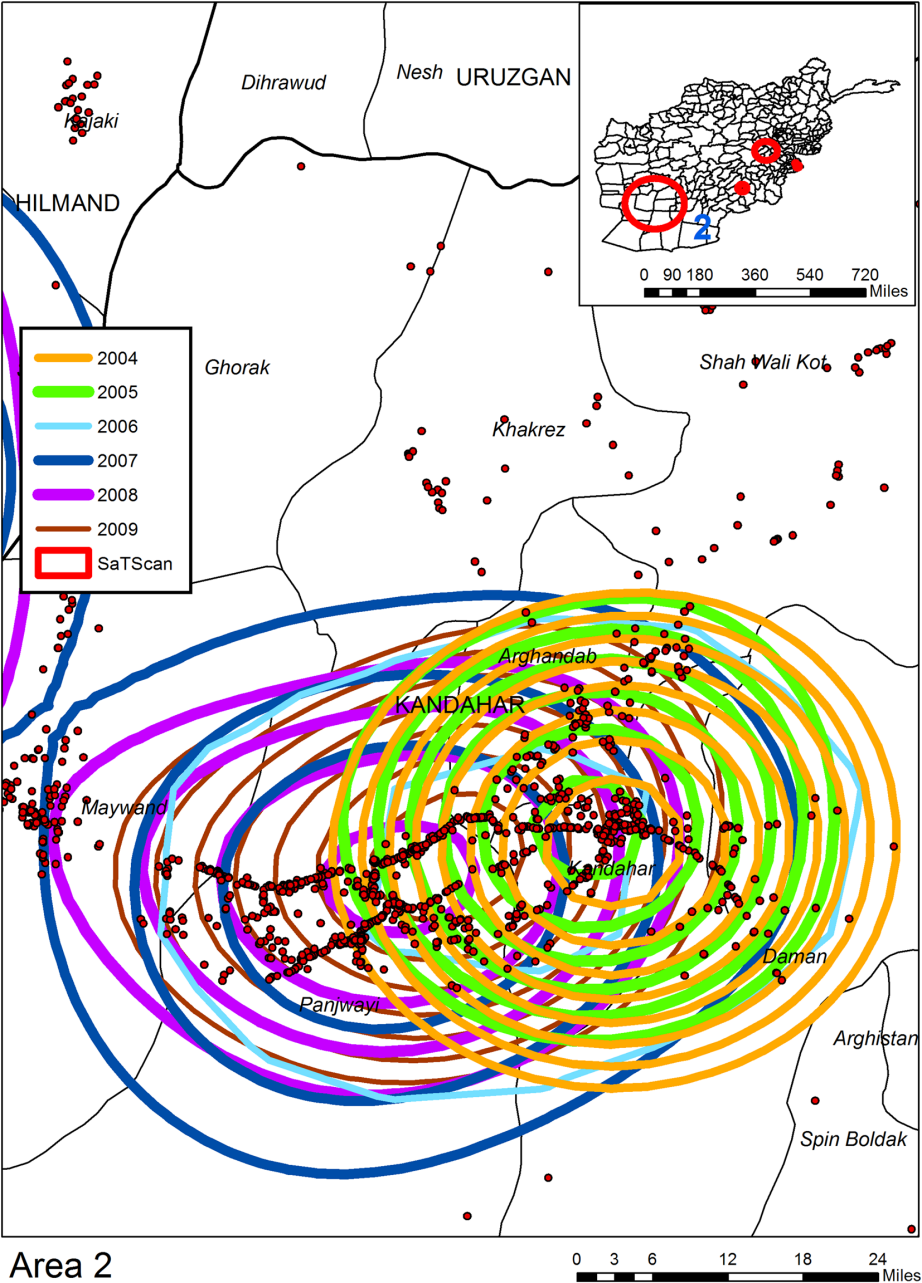
KDE and SaTScan analyses of IEDs for Area 2 (Kandahar Province)

**Fig. 3:**
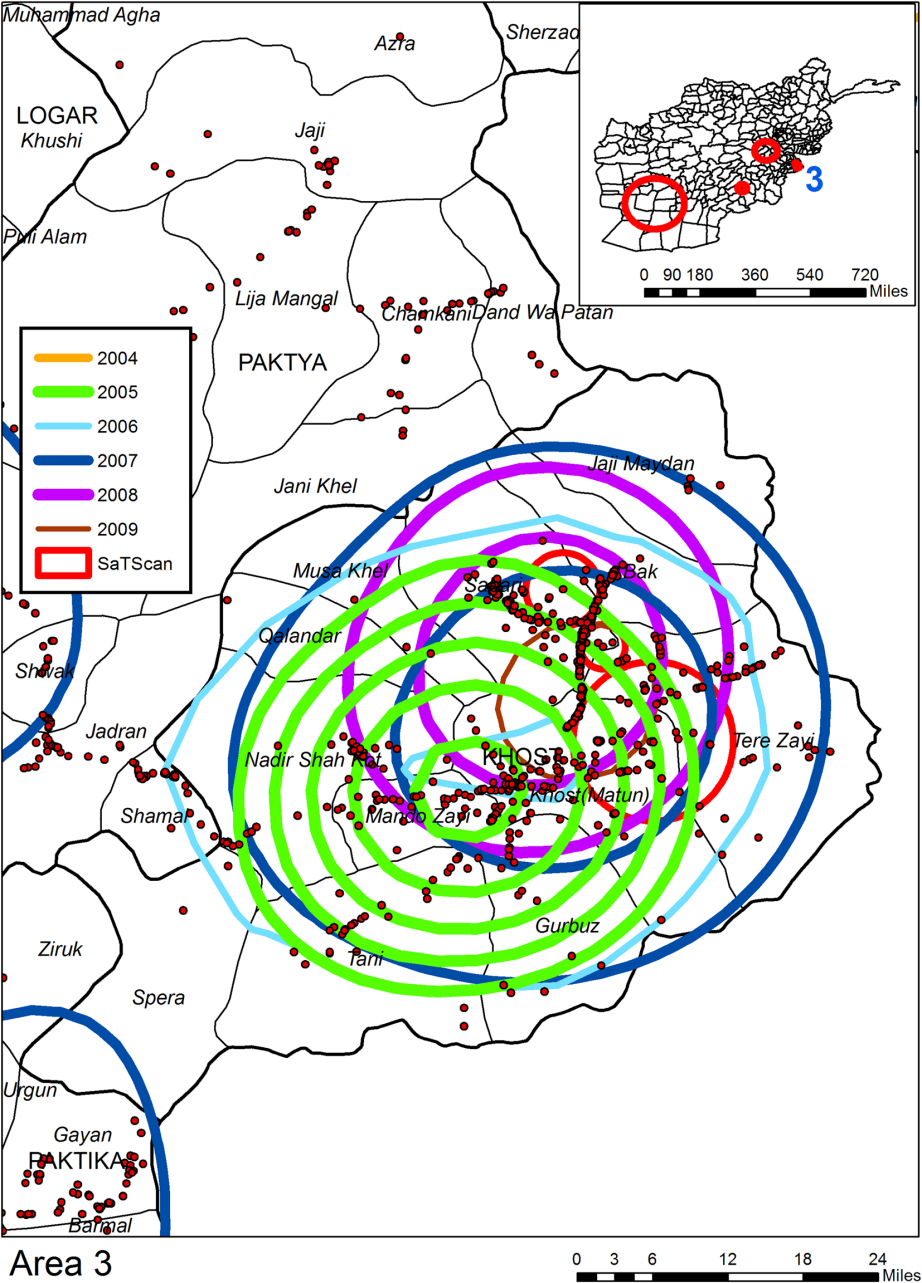
KDE and SaTScan analyses of IEDs for Area 3 (Khost Province)

Six KDE are applied to IED data extracted from the AWD, one for each year for the period 2004–2009.^10^ Each year’s KDE is classified into ten even breaks with only the top 50 % of the density map being displayed (as contours). By overlapping the contours for all years, composite intensity maps are created and from these six “hotspot”^11^ regions are identified. For the purpose of reporting in Table 1 each hotspot is buffered outwards by approximately 10 miles in order to reduce the impact of imposing an artificial boundary on the data. All IEDs within this buffer boundary are counted for each year, with hotspot years being shown in red. The next three figures show the geographic overlay of all KDEs, along with the SaTScan result (described in a following section), and the locations of all IEDs. Each annual KDE contour is displayed as a different color. In this way results can be visually compared across the years, with the underlying pattern of IEDs showing micro patterns of concentrations. Figure 1 shows Area 1 (Helmand Province); what is immediately evident is that this area becomes more violent later in the time sequence, with IED hotspots being visible from 2007 to 2009. By comparison Area 2 in Fig. 2 (Kandahar) displays a consistent pattern of attack across the 6 year period, with the total number of IEDs also increasing each year. Area 3 in Fig. 3 (Khost Province) also produces IED hotspots for every year except 2004, though the overall percentage rise in the total number of IEDs between the years is lower than for Areas 1 and 2. Areas 4 to 6 (not displayed) have less consistent patterns. Indeed, the number of IEDs in Area 6 (Kunar Province) actually decreases in 2009.

**Table 1:** Number of IEDs falling in visibly determined KDE hotspots

	2004	2005	2006	2007	2008	2009
Area 1	3	12	36	*141*	*274*	*541*
Area 2	*18*	*32*	*99*	*143*	*242*	*504*
Area 3	7	*28*	*81*	*91*	*160*	*212*
Area 4	2	7	30	*31*	15	30
Area 5	*17*	*21*	25	30	25	60
Area 6	*16*	*17*	25	25	27	21

Italic values indicate the total number of IEDs falling inside the area common to each year’s KDE highest contour when overlaid together

**Fig. 4:**
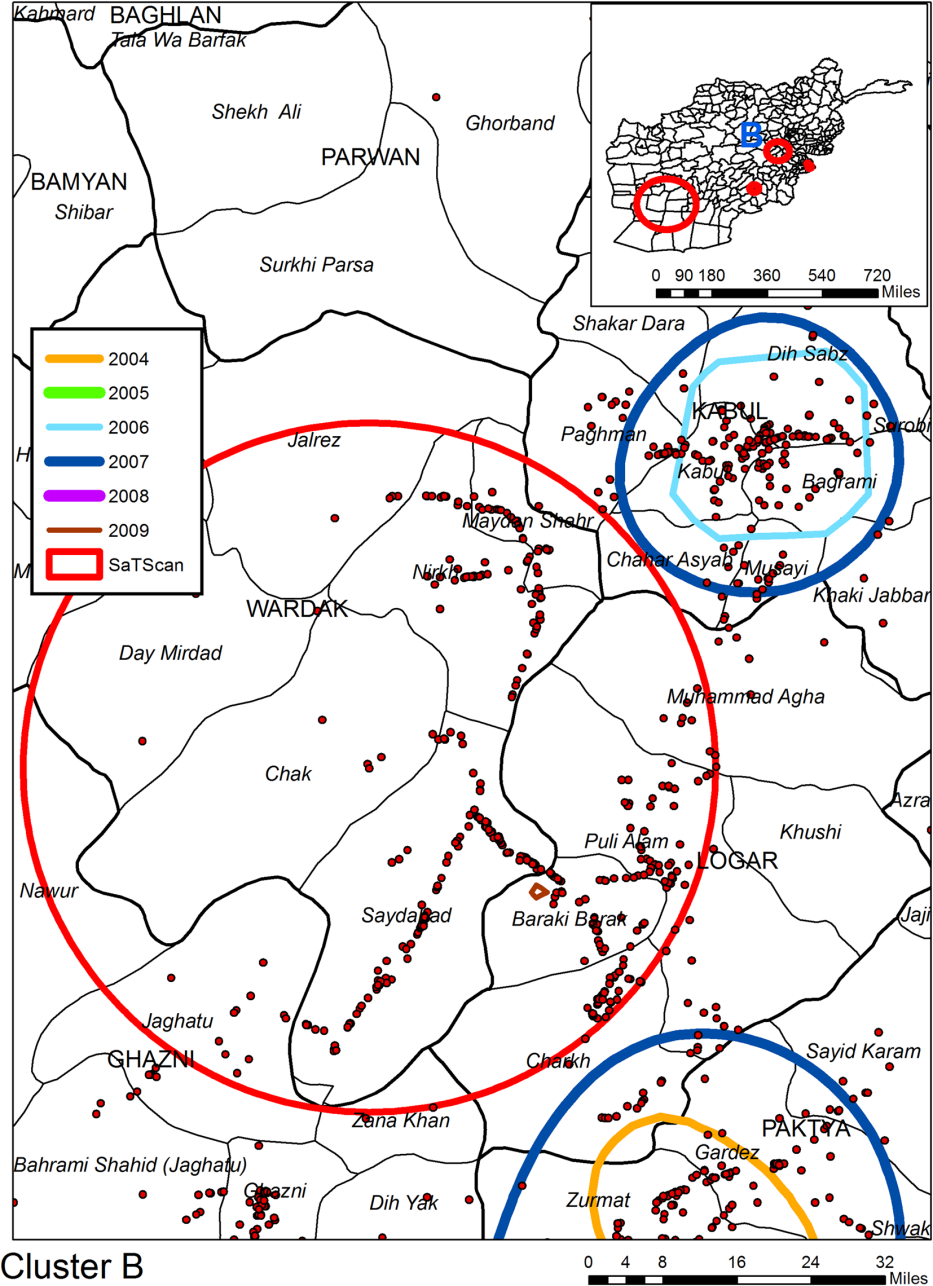
KDE and SaTScan analyses of IEDs around Cluster B

The second space time analysis uses SaTScan to identify patterns of IEDs by day. There are six SaTScan clusters which can be seen on the inset maps but only three of these are statistically significant (see Table 2 and the red circles in Figs. 1, 2, 3). Cluster “A”, the edge of which can be seen in Fig. 1 is the largest; Cluster “B” is the second largest circle on the inset maps displayed on Fig. 4. Interestingly enough this cluster does not overlay with any of the KDE hotspots, and even the most proximate contours are for 2006 and 2007 to the northwest and 2004 and 2007 to the southwest. Cluster “C” is shown in Fig. 3 along with two further non-significant SaTScan clusters. For all three of the SaTScan significant clusters the time component occurs within 2009. One interpretation could be even within the increasing trend of attacks, there was still a more strategized plan of attack for 2009.

**Table 2:** Statistically significant space–time clusters identified using SaTScan

Cluster	Radius (km)	IEDs	Time frame	P value
A	132.79	668	2009/6/14 to 2009/12/31	1.00E−17
B	49.73	302	2009/3/21 to 2009/12/31	1.80E−13
C	8.56	27	2009/10/15 to 2009/12/31	0.0011

**Fig. 5:**
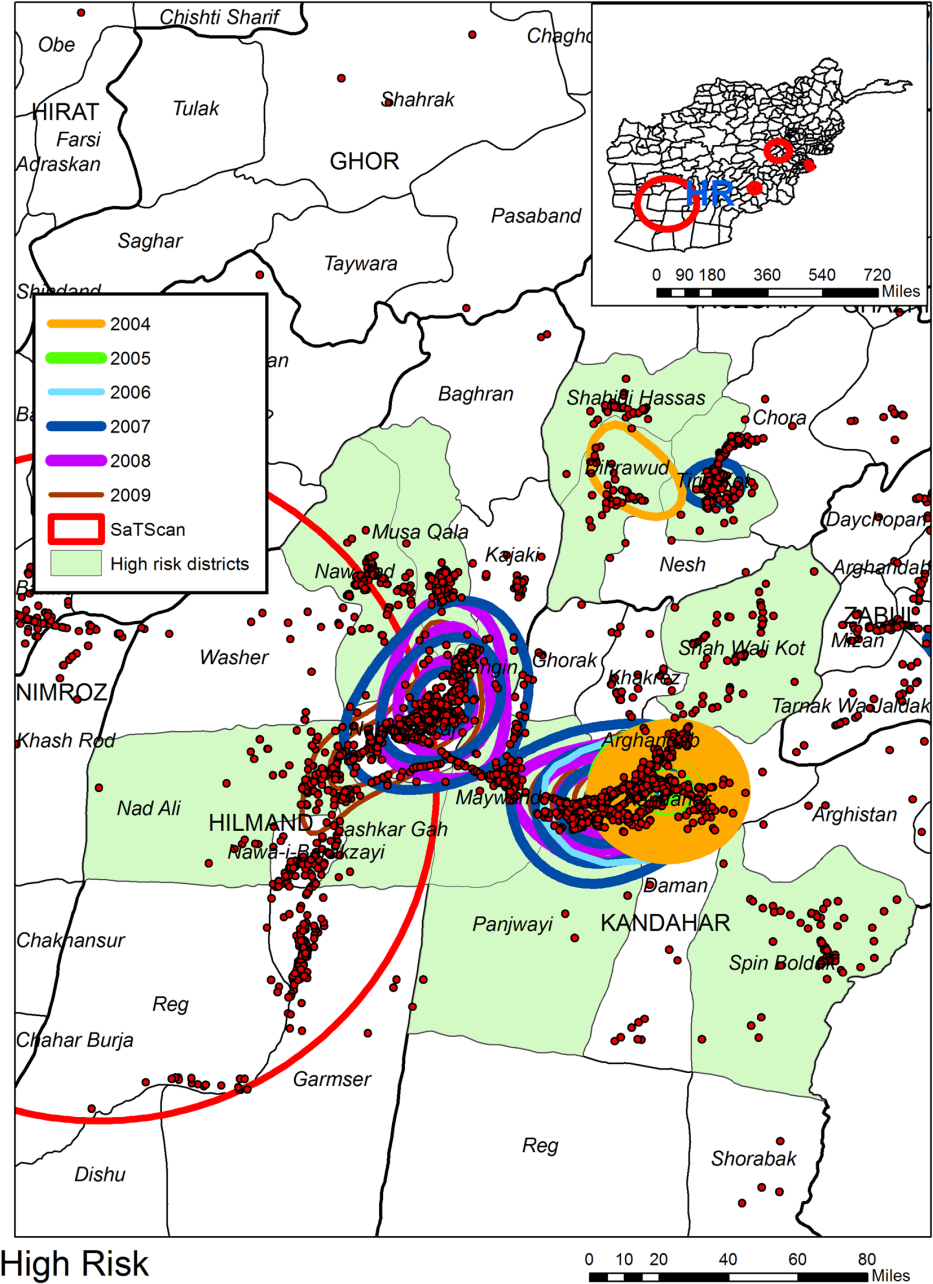
KDE and SaTScan IEDs results overlaid on Districts commonly associated as having high levels of violence

Figures 1, 2, 3 and 4 also illustrates the spatial heterogeneity of IEDs even within the hotspots. This is particularly evident along transportation routes and most easily seen in Figs. 2 and 4. Although not displayed on the maps to reduce clutter, there is also heterogeneity in IED patterns around settlements; there is a high intensity for some (seen as a centralized intensity of IED points on Fig. 1 for example), whereas other settlements are more isolated both in terms of violence and transportation connectivity. Therefore, any health outcome reported by a District aggregation is likely to over count disease risk for more isolated settlements, and undercount risk in proximity to roads and violence “hotspot” settlements. This heterogeneity can be seen in Fig. 5 as spatially varying concentration of points within the shaded districts which are commonly identified as being violent. A boundary effect is also evident for both the KDE and SaTScan hotspots which cover multiple districts. These aggregation and boundary effects are well known to geographers, though it is not as a common to have access to a data set, usually because of confidentiality concerns, where these problems can so easily be visualized and disseminated.

## Discussion

The release of the WikiLeaks AWD provides an interesting opportunity for the analysis of violence in Afghanistan for the period 2004–2009. These data also offer a unique opportunity to analyze the conflict-health nexus. One example of where these data can contribute to a better understanding of this relationship is the impact on polio, particularly because of the challenges violence poses to regional vaccination schemes. Unfortunately comparable fine scale polio surveillance data is hard to acquire, yet this does not stop us considering the likely impacts on vaccine delivery and disease based on the spatial patterns of IEDs. Consistent with other findings [6] we found a general increase in IED attacks with each successive year but this trend is not geographically homogenous, and even declines in Area 6. From a health perspective, given the increasing intensity of violence in Area 1, if we accept that health is negatively associated with conflict, we would expect the disease burden to be worse in 2007–2009 compared to 2004–2006. Of course this relationship is actually more complex with different temporal lags occurring with different diseases. If we accept our previously described finding of a 2 year lag between violence and an increase in polio then polio disease risk would extend to 2011. The situation in Area 2 is even more problematic given the consistently high levels of violence. What our maps show, however, is the geographically focused nature of these attacks around transportation arteries. This might cause additional complexity to the violence-health nexus as there is likely to be an impact on the mobility of health services, including vaccine deliveries, to areas not affected by the violence.

Although the results of the SaTScan analysis are interesting from a space–time data analysis perspective, the selection of a fine temporal unit (1 day), in combination with increasing levels of violence in 2009 makes this a less appropriate approach for epidemiological comparison. Apart from the technique being harder to run, requiring programing skills to manipulate data for input into the model, and using considerably more computing power, the final results are not as revealing as the KDE. In particular the primary cluster covers multiple district areas, and is only relevant for 2009, even though we know there were other spatial patterns of violence across all the years. We acknowledge that these results might be due to a combination of the temporal aggregation chosen and the increase in IED intensity for 2009.^12^ Even though further work is needed to look at how SaTScan should be best utilized on the AWD, we feel comfortable in suggesting a KDE (or similar style) visualization of these data are the most useful if results are to be compared against other health data. This is important as most intermediate GIS analysts should feel comfortable in running such a technique.

We have shown that there is considerable IED geographic and temporal heterogeneity. The next question is how best to leverage these findings (and more importantly these data), into other epidemiological investigations. In this way we might be able to explore more spatially specific questions, such as does the distance decay function vary for different areas and diseases with regards a conflict-health relationship?^13^

**Fig. 6:**
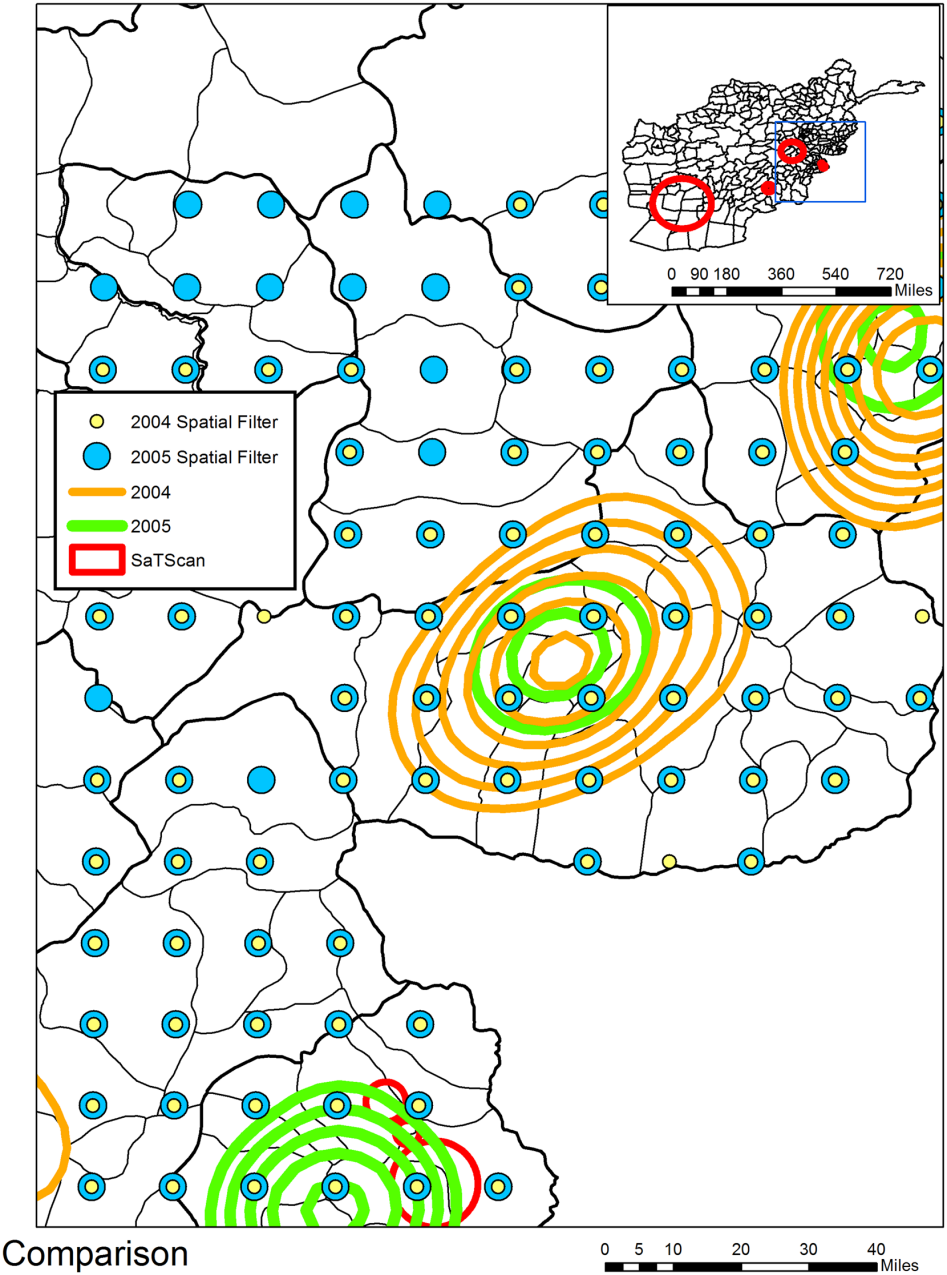
Spatial Filter of IED rates for 2004 and 2005 coinciding with a high risk of malaria

**Fig. 7:**
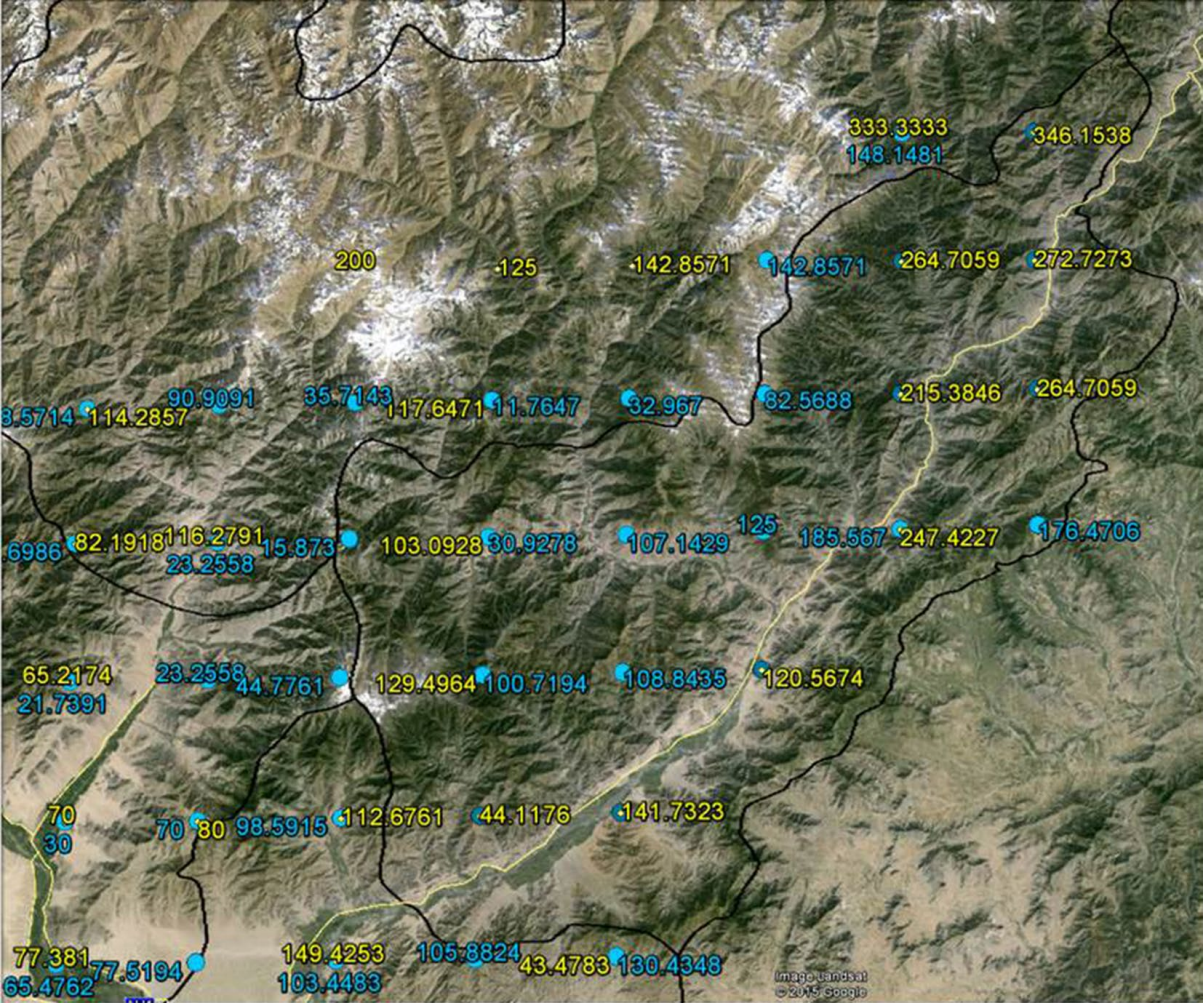
Example of IED Spatial Filter rates exported to Google Earth coinciding with a predicted high rate of malaria

As an example of how such subsequent research can develop, consider Brooker and colleagues work on endemic *Plasmodium vivax* malaria risk which incorporated a survey of 269 villages in 2005 supplemented with an additional survey of 333 villages between 2000 and 2003 [10]. The authors mention the importance of violence, and in fact comment that the previous fall of the Taliban during 2001 had led to improved malaria control and delivery, but violence is not used as an input into their simulation models. By spatially overlaying their detailed publication maps with the hotspots generated from our KDE analysis, visual connections can be made as to why this might be a fruitful avenue of investigation. However, in order for subsequent research to be made more likely, it is important to release data and maps similar to our KDE output that are easily available. To this end we also conducted a spatial filer analysis for two major areas of Afghanistan based on the overlay of all KDE results.^14^ For both areas a bounding box was created, and a grid overlaid inside in order to create a smoothed overlapping rate map of IED explosions. Three decisions were made in the calculation of this rate; the size of the filter (in this case 20 miles though other radii were also analyzed), choosing the numerator as being all IED locations per year, and the denominator which were all IED locations for the entire period of 2004–2009. A modified version of DMAP [28] was used to calculate the spatial filter surface. The resulting output, once manipulated into a GIS environment, is a grid of nodes where each location is the annual rate of IEDs within 20 miles. The benefit to other researchers is that once this rate map is overlaid with other spatial data, through a spatial join the proximate rate for any key location can be attached and then included in analyses. The second area (seen in the inset map of Fig. 6) spatial filter is relevant to the region of Afghanistan highlighted by Brooker and colleagues as having the highest incidence of malaria. Figure 6 also shows the IED KDE hotspot for 2004 and 2005; a comparable time frame with their village data collection. Finally, this figure also shows the spatial filter nodes for both 2004 and 2005 that generated an IED rate. In order to maximize the transferability of the IED map to other researchers, all spatial filters were exported into Google Earth. Figure 7 shows the outline of Kunar province which had the highest predicted probability (especially along the main road) of *P. Vivax* transmission based on Brooker and colleague’s logistic regression model. The associated spatial filter grids display the rate of IEDs (per 1000 IEDs) for 2004 (yellow), and 2005 (light blue) for this predicted area. Even from this graphic we can see the northeastern trend in violence, which becomes more pronounced in 2005. It would be easy to analyze the village surveys by spatially joining each to their closest IED node.^15^

## Conclusion

Even though the release of the AWD generated considerable debate as to whether ethically researchers should use it, and concerns were raised that analyses could potentially reveal military strategies that might impact current operations [7], we believe a counter narrative is that conflict data such as these can help in predicting health outcomes, and should certainly be included in predictive modeling. Theoretical developments from such analyses are not only important in understanding the historical context, but also in helping explain how current ongoing conflict situations will affect the health of those in proximity. Although one argument is the AWD represent only one location and for one time period, similar data are still being collected and we believe there is a health justification to create a standardized fine scale release. This paper also suggests at the additional possibilities for research if more health data are collected, or made available, at finer spatial scales. Other countries report data (such as polio) at a far finer granularity than an Afghan District. It would be useful for the World Health Organization to address this issue of inconsistent data availability, even given the impediments occurring through the local political situation. Even without a standard release, we have shown a data resource that can be made available to researchers who have access to finer scale health data.

Although some of the previously described papers which have modeled explosive devices offer more insight with regards potential covariates than the relatively simple KDE and spatial filtering approaches described here, we see this paper as a first step. What we have done here is add a detailed spatial layer of a violence proxy that can be analyzed in conjunction with health data—even this relatively simple combination has previously not been available but rather left as anecdote or for the discussion section of a report or academic article.

At the time of writing, the situation in Syria and Iraq provide two country examples where such data could help improve our theoretical understanding, and could help shape intervention, response and political will.

## Footnotes

1 We include literature with regards Iraq due to the overlap in similar data input, techniques used, and general research context.

2 It should be remembered that surveillance data quality is often questionable in conflict areas though it is suggested that as reporting exceeded the WHO expected incidence of 1–2 cases per 100,000 in every Afghan region it can be considered reliable. The reader should also remember that these data initially exist at a fine granularity but are aggregated to the District for reporting purposes. Although there are potentially near-real time safety consequences with releasing these data—it could be argued that after an agreed upon temporal buffer, researchers should be allowed access to this rich primary data source.

3 Unpublished research by the authors which acted as a conceptual precursor to the work presented here.

4 It should be noted that the physical location of an IED is only one of several possible attributes that can weight an event. For example, the number of deaths, injuries, or the mortality ratio. Others have found that there is less pattern with the number of mortalities as these to some degree are random—although there is an association with the effectiveness of the device, but the unknown is how many potential victims are within range at the time of explosion [25].

5 For example, Medina and colleagues used a temporal unit of 6 months in their analysis ibid.

6 It should be noted that others have chosen other more disaggregate classification schemes so as to limit artificial breaks and produce more visually appealing color transformations from high to low intensities on the map [20], though the type of classification applied here is the most common means of visualization.

7 Secondary clusters with geographic overlap were not considered.

8 Benigni and Furrer [7] suggested daily activity as an appropriate time frame because of diurnal patterns matching military operations, while Zammit-Mangion et al. [39] used a weekly aggregation when analyzing the AWD data.

9 One consideration for fine temporal aggregations is the computing complexity required for the analysis of such large datasets. Running SaTScan on daily data from the AWD took considerable desktop PC performance; over 272 h on 8 processors.

10 Other temporal aggregations were also analyzed, some of which might be more relevant to different disease systems. Though for space considerations, only annual analyses are reported here.

11 We understand our use of the phrase “hotspot” is liberal and only denotes the areas of high concentration as identified by KDE. It is used more for ease of description than as a statistical expression.

12 Further research needs to vary both the temporal aggregations and the bounded area being analyzed. In this regard, these data provide an excellent source to explore the limitations of this and other space time analyses.

13 This type of catastrophic event-health distance banding has been performed in the United States, for example in association with the attacks in New York City on September 11th, 2001.

14 Two areas were preferred that encompassed most of the IED events—a spatial filter grid for the entire country would not bare any more insight and would take considerably more computing effort for the type of fine scale filters we were applying.

15 The spatial filter grids are available from the authors as KMZ files at different spatial and temporal resolutions depending on the health situation to be analyzed. Please contact the lead author for details.
